# ProQ3: Improved model quality assessments using Rosetta energy terms

**DOI:** 10.1038/srep33509

**Published:** 2016-10-04

**Authors:** Karolis Uziela, Nanjiang Shu, Björn Wallner, Arne Elofsson

**Affiliations:** 1 Department of Biochemistry and Biophysics and Science for Life Laboratory, Stockholm University, 171 21 Solna, Sweden; 2 Bioinformatics Short-term Support and Infrastructure (BILS), Science for Life Laboratory, 171 21 Solna, Sweden; 3 Department of Physics, Chemistry and Biology (IFM)/Bioinformatics. Linköping University, 581 83 Linköping, Sweden

## Abstract

Quality assessment of protein models using no other information than the structure of the model itself has been shown to be useful for structure prediction. Here, we introduce two novel methods, ProQRosFA and ProQRosCen, inspired by the state-of-art method ProQ2, but using a completely different description of a protein model. ProQ2 uses contacts and other features calculated from a model, while the new predictors are based on Rosetta energies: ProQRosFA uses the full-atom energy function that takes into account all atoms, while ProQRosCen uses the coarse-grained centroid energy function. The two new predictors also include residue conservation and terms corresponding to the agreement of a model with predicted secondary structure and surface area, as in ProQ2. We show that the performance of these predictors is on par with ProQ2 and significantly better than all other model quality assessment programs. Furthermore, we show that combining the input features from all three predictors, the resulting predictor ProQ3 performs better than any of the individual methods. ProQ3, ProQRosFA and ProQRosCen are freely available both as a webserver and stand-alone programs at http://proq3.bioinfo.se/.

Protein Model Quality Assessment (MQA) has a long history in protein structure prediction. Ideally, if we could accurately describe the free energy of a protein, this free energy should have a minimum at its native structure. Methods to estimate free energies of protein models have been developed for more than 20 years^1^,^2^,^3^. These methods are focused on identifying the native structure among a set of decoys and therefore not necessarily have a good correlation with the relative quality of protein models.

In 2003 we developed ProQ that had a different aim than earlier methods^4^. Instead of recognising the native structure, the aim of ProQ is to predict the quality of a protein model. ProQ uses a machine learning approach based on a number of features calculated from a protein model. These features include agreement with secondary structure, number and types of atom-atom and residue-residue contacts. One important reason for the good performance of ProQ is that each type of contacts, both atom- and residue-based ones, is normalised by the total number of contacts as in Errat^5^.

In the first version of ProQ the model quality was estimated for the entire model. In 2006 we extended ProQ so that we estimated the quality of each residue in a protein model, and then we estimated the quality of the entire model by simply summing up the quality for each residue^6^. This method was shown to be rather successful in CASP7^7^ and CASP8^8^.

In comparison to other methods, ProQ performed quite well for almost a decade, but some five years ago one of us developed the successor, ProQ2^9^. The most important reason for the improved performance of ProQ2 was the use of profile weights, and features averaged over the entire model even though the prediction was local. ProQ2 has since its introduction remained the superior single model based quality assessor in CASP^10^.

In CASP it has also been shown that the consensus type of quality estimator is clearly superior to the single-model predictors. Consensus estimators are based on the Pcons approach that we introduced in CASP5^11^,^12^. In these methods, the quality of a model, or a residue, is estimated by comparing how similar it is to models generated by other methods. The idea is that if a protein model is similar to other protein models, it is more likely to be correct. The basis of these methods is a pairwise comparison of a large set of protein models generated for each target. Various methods have been developed but the simplest methods such as 3D-Jury^13^ and Pcons^14^ are still among the best.

A third group of quality assessors also exist, the so-called quasi-single methods^15^. These methods take a single model as an input and compare its similarity with a group of models that were built internally.

It has been clear since CASP7 that quality assessment with consensus methods is superior to any other quality assessment method^7^. However, it has lately been realised that these methods have their limitations^10^. Consensus methods and quasi-single methods appear not to be better than single-model based models at identifying the best possible model. In particular, when there is one outstanding model, as the Baker model for target T0806 in CASP11^16^, the consensus-based methods completely fail, but the single model methods succeed^10^. Furthermore, a consensus based quality predictor cannot be used to refine a model or be used for sampling. Finally, single-model methods can be used in combination with consensus methods to achieve a better performance than either of the approaches^10^. Therefore, the development of improved single-model quality assessors is still needed.

Here we present two novel single-model predictors, ProQRosCen and ProQRosFA, which are based on Rosetta energy functions. In addition, we present the third novel predictor ProQ3, which combines training features from ProQRosCen, ProQRosFA and ProQ2.

## Results and Discussion

In this section, we describe the most important aspects of our method development, which might give some insight for others working on the same problem. Thereafter, we move on to benchmark the novel predictors. The more technical details of our method implementation will be covered later in the Methods section.

### Method development

ProQ2 is a machine learning method based on Support Vector Machines (SVM) that was recently implemented as a scoring function in Rosetta^17^. ProQ2 uses a variety of input features, including atom-atom contacts, residue-residue contacts, surface area accessibilities, predicted and observed secondary structure and residue conservation to predict the local residue quality. A general problem when selecting input features for machine learning methods is that they should be independent on protein size and other protein specific features, i.e. they need to be normalised in a proper way. In ProQ2 this is done by describing contacts of a particular type as fractions of all contacts.

The new predictors are based on different input features but trained in a similar way as ProQ2. The input features are Rosetta^18^ energy terms. Rosetta uses two energy functions: one based on all-atoms (“full-atom” model) and one that uses a simplified centroid side-chain representation (“centroid” model). In general, the all-atom function provided more accurate energies, but the centroid function is useful when an all-atom model is not available or when the model is created using a different force field, since it is less sensitive to exact atomic details. Therefore, we developed two new predictors: one that uses full-atom model (“ProQRosFA”) and one that uses centroid model (“ProQRosCen”). In addition, we developed a third predictor that combines ProQRosFA, ProQRosCen and ProQ2 (“ProQ3”).

The new predictors use the same method to train a linear SVM as was used in ProQ2. Here the quality of each residue is described using the S-score^19^,^20^ and used as a target function. However, the descriptions of the local environment surrounding a residue are completely different in the new predictors.

#### ProQRosFA input features

For the predictor ProQRosFA, we used “talaris2013” weight set that is currently the default energy function in Rosetta and consists of 16 energy terms that are summed up to form the total Rosetta energy score. First, we examined how well each energy term correlates with the local model quality as measured by our target function (S-score) on the CASP11 data set. A stronger correlation between an input feature and the target function is more useful for the final predictor. Since there are many individual input features, rather than showing the correlation for each individual feature, we grouped them into seven groups and show the correlations for each group:

- **Van der Waals**: fa_atr, fa_rep, fa_intra_rep
- **Solvation**: fa_sol
- **Electrostatics**: fa_elec
- **Side-chains**: pro_close, dslf_fa13, fa_dun, ref
- **H-bonds (Hydrogen bonds)**: hbond_sr_bb, hbond_lr_bb, hbond_bb_sc, hbond_sc
- **Backbone**: rama, omega, p_aa_pp
- **Total-energy-FA**: score

The last group (Total-energy-FA) is a sum of all energy terms used in the ProQRosFA predictor with weights taken from the “talaris2013” function. Note that even though we grouped features here for visualising their performance, they were all used separately when training the final SVM.

**Figure 1:**
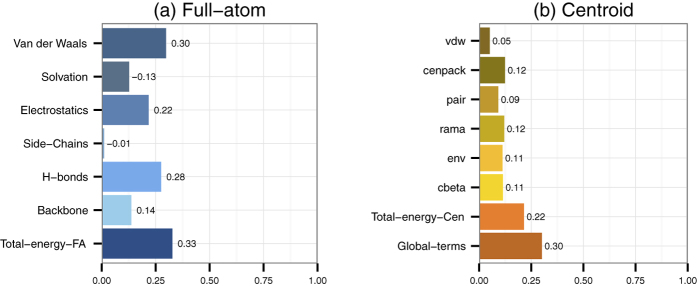
Spearman correlations of full-atom (**a**) and centroid (**b**) Rosetta energy terms against the target function (S-score). All correlations are calculated on the local (residue) level. Total-energy-FA and Total-energy-Cen are the sums of all local full-atom and centroid energy terms. Global-term is the sum of all global centroid energy terms that are not shown in the plot (*rg*, *hs_pair*, *ss_pair*, *sheet*, *rsigma*, *co*). Negative correlations (Solvation and Side-Chains) are shown with a positive bar length. Test set: CASP11.

Figure 1a shows Spearman correlations against our target function (S-score) for each of the seven groups. The correlations for Van der Waals, Electrostatics, Hydrogen bond and Total-energy-FA groups are higher than for Solvation, Side-Chains and Backbone. In general, solvation is the main driving force for protein folding but here it actually has a negative correlation with model quality, i.e. better models do in general have worse solvation energy, highlighting that the problem of quality estimation is different from estimating the free energy of a native structure. Anyhow, the Total-energy-FA group including all the features shows the highest correlation even if the difference to Van der Waals and H-bonds is small.

#### ProQRosCen input features

Centroid scoring functions have an advantage that they can be used even if the exact position of a side chain in the model is not known. They are also less sensitive to exact atomic positions that make them possible to score models from different methods with a lower risk of high repulsive score from steric clashes.

For the predictor ProQRosCen, we used all energy terms from the standard centroid scoring function “cen_std”—*vdw*, *pair*, *env* and *cbeta*. In addition to that, we included two more centroid energy terms that were not part of “cen_std” function—*cenpack* and *rama*. The term Total-energy-Cen is defined as the sum of all of the above centroid energy terms including *cenpack* and *rama*.

The scoring functions “talaris2013” and “cen_std” include only **local** energy terms. However, there are also potential useful **global** energy terms that are defined for the whole protein model. Here we included six global centroid energy terms in our ProQRosCen predictor: *rg* (radius of gyration of centroids), *co* (contact order), and statistical potential terms for secondary structure formation: *hs_pair*, *ss_pair*, *sheet*, *rsigma*. For simplicity, we only show the correlation for the sum of all of these global energy terms (Global-terms in Fig. 1b).

Most of the full-atom energy groups correlate better than the individual centroid energy terms. Also, we can see that the correlation for Total-energy-FA is higher than the correlation of the Total-energy-Cen. Finally, it can be noted that the global centroid energy terms are clearly performing better than the local centroid energy terms, although these terms predict the same quality (energy) to all residues within a model.

#### Training an SVM and using averaging windows increases the performance

**Figure 2:**
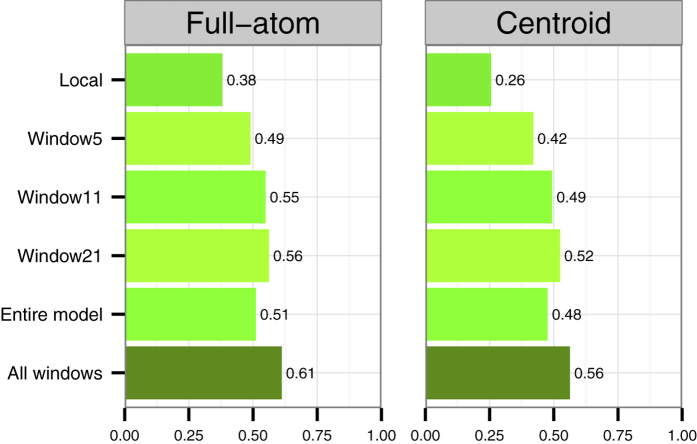
Spearman correlations of SVM predictions against the S-score using different window sizes to average full-atom and centroid energy terms that are used as input features. All correlations are calculated on the local (residue) level. Only local centroid energy terms are included, because global energy terms cannot be averaged over different window sizes. Training set: CASP9. Test set: CASP11.

A straightforward approach to use the energy terms for predicting the local quality is to train an SVM using all Rosetta energy terms corresponding to that residue. The correlation of the original Rosetta energy functions with model quality is 0.33/0.22 for the full-atom/centroid models respectively (see Fig. 1). However, if all the individual energy terms are used as inputs to an SVM the performance increases to 0.38/0.26 (see Fig. 2, Local).

Further, we notice that we can improve the prediction performance by calculating the average energy over windows of varying size before training the SVM. Figure 2 shows the impact of window sizes on the prediction performance. In general, even a small window provides a substantial improvement, but larger windows result in a better performance. If we use a window of 21 residues to average the input energy terms, the correlations increase to 0.56 and 0.52 for full-atom and centroid predictors, respectively. However, if we take it to the extreme and use a window that covers the entire model, the correlations drop slightly.

**Figure 3:**
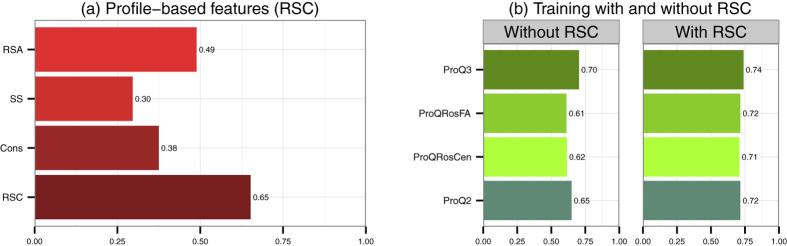
(**a**) Spearman correlations of profile-based features (RSA, SS and Cons) and their combination (RSC) against the target value (S-score). RSA, SS and Cons are taken as raw values without using SVM, but RSA and SS are averaged over a window of 21 residues. RSC combines RSA, SS and Cons using SVM with 3 different window sizes, as in ProQ2 (see Methods). (**b**) Spearman correlations of ProQ3, ProQRosFA, ProQRosCen and ProQ2 against the S-score with and without including RSC (RSA, SS and Cons) into the training. Here, ProQRosCen includes both local and global energy terms. Training set: CASP9. Test set: CASP11.

Next, we noticed that the combination of several window sizes as input to the SVM provides the best results. When we combine all the window sizes, the correlation reaches 0.61 for the full-atom predictor, and 0.56 for the centroid predictor. When adding the global centroid terms to the centroid predictor the correlation increases to 0.62, see Fig. 3b.

#### Profile-based features

The only type of features that are common between ProQ2, ProQRosFA and ProQRosCen are the profile-based features: Relative Surface Area accessibility agreement (RSA), Secondary Structure agreement (SS) and Conservation (Cons). We refer to these features as profile-based, because they are based on information that can be extracted from a sequence profile. Two features, RSA and SS, indicate the agreement between predicted and observed RSA/SS values (see Methods). The third feature, conservation, depends only on the sequence profile and has the same values for all of the protein models from the same target. We refer to these features as RSC (RSA, SS, and Cons), see Fig. 3b.

We would like to emphasise that the profile-based features are essential in model quality assessment. As we can see from Fig. 3a, these features alone without training provide reasonable correlations with the target value. When we train an SVM to predict the local quality using only RSA, SS and Cons as an input, we reach correlation as high as 0.65. That is the same correlation as for all other features in ProQ2 excluding RSC (see Fig. 3b) but when we combine them, the correlation only increases to 0.72 (Fig. 3a,b). The correlation for ProQRosFA, ProQRosCen and ProQ2 also improves when adding RSC.

In general, we noticed that it is relatively easy to reach a correlation of around 0.60–0.65, but it appears to be difficult to increase it further. The original ProQ2, ProQRosFA, ProQRosCen and RSC all obtain correlations of 0.60–0.65. Only by combining the input features from all of the predictors we reach a correlation of 0.70 without RSC and to 0.74 with RSC. Although this improvement is small it is still significant using the Fisher r-to-z transform that accounts for the fact that the correlation coefficient distribution is negatively skewed for larger correlation values (>0.4).

Although our goal was to develop novel predictors that use different input features than ProQ2, we still included profile-based features into ProQRosFA, ProQRosCen. Similar profile-based features are not only used in ProQ2, but also in many other model quality assessment methods^21^,^22^,^23^. We can see that these features are important for the predictor’s performance and they almost become de-facto standard in single-model methods. Therefore, it was interesting to compare ProQRosFA and ProQRosCen performance with other methods after including these features.

### Benchmark

In this section, we compare the newly developed methods ProQRosFA, ProQRosCen and ProQ3 with their predecessor ProQ2 and other publicly available single-model methods: QMEAN^23^, Qprob^22^, SMOQ^24^, DOPE^25^, dDFIRE^26^ on the CASP11 and CAMEO^27^ data sets (see Methods). We compare the method performance in three categories: local (residue) level correlations, global (protein) level correlations and model selection. Two of the methods (Qprob and dDFIRE) provide only the global level predictions, so they are not included into the local level evaluation.

#### Local correlations

All of the new predictors (ProQRosFA, ProQRosCen and ProQ3) are trained on the local level, i.e. the quality is estimated for each residue independently. Therefore, the correlation with the target value on the local (residue) level is examined first.

**Figure 4:**
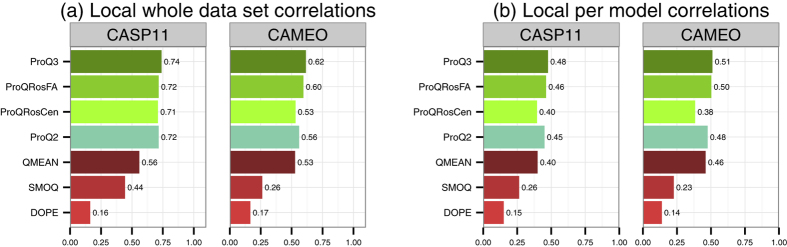
Spearman correlations of QA methods against the S-score on local (residue) level. (**a**) Correlations for the whole data set (**b**) Average correlations for each model in the data set.

We evaluated all methods in two categories: first the correlation over the whole data set (Fig. 4a) and secondly the average correlation calculated for each model in the data set (Fig. 4b). The first category of evaluation shows how well methods separate between well- and badly-modelled residues in general while the second shows how well methods separate well- and badly-modelled residues within a particular model.

ProQ3 outperforms all other single-model methods on both data sets and in both categories of evaluation. The largest improvement over ProQ2 is found in the CAMEO for whole data set correlation (0.62 vs. 0.56). ProQRosFA performs equally or slightly better than the original ProQ2 while ProQRosCen performs slightly worse, but still on par with QMEAN. Both QMEAN and DOPE perform equally or worse than any ProQ method with the only exception of QMEAN having a higher per model correlation than ProQRosCen in the CAMEO data set (0.46 vs. 0.38, Fig. 4b).

All differences in local whole data set correlations (Fig. 4a) are significant with P-values <10^−3^ according to Fisher r-to-z transformation test. All differences in mean per model correlations were significant with P-values <10^−3^ according to Wilcoxon signed-rank test.

#### Global correlations

Even though ProQRosFA, ProQRosCen and ProQ3 are trained on the local level, they also provide global predictions of the quality of a model. The global predictions are derived from the local predictions, by summing up all local predictions for a protein model and then dividing the sum by the target protein length. The target function (S-score) is also local by its nature, but can be converted to global in exactly the same way.

**Figure 5:**
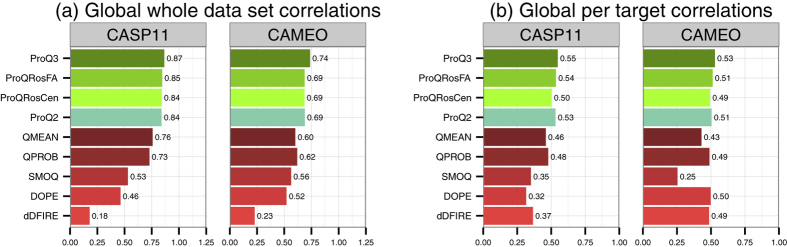
Spearman correlations of QA methods against the S-score on global (protein) level. (**a**) Correlations for the whole data set (**b**) Average correlations for each target in the data set.

We evaluated all methods again in two categories: the first is the correlation over the whole data set (Fig. 5a) and the second is the average correlation calculated for each target in the data set (Fig. 5b). The first category shows how well a method separates good and bad models in general, while the second shows how well a method separates good and bad models for the same target.

ProQ3 again outperforms all other single-model methods on both data sets and in both categories of evaluation. The largest improvement over the original ProQ2 is in the CAMEO whole data set correlation (0.74 vs. 0.69), Fig. 5a. In the whole data set evaluation category, both ProQRosFA and ProQRosCen performance is close to ProQ2 and better than the rest of the methods. In the per target evaluation category, ProQ methods still outperform all the rest on CASP11 data set, but on CAMEO data set the differences are small. The reason for this is that in the CAMEO data set the model quality within a target varies much less (see Table 2).

All differences in the global whole data set correlations (Fig. 5a) are significant with P-values <10^−3^ according to Fisher r-to-z transformation test. Per target correlation differences are not significant within ProQ methods with the only exception of ProQ3 performing significantly better than ProQRosCen on CAMEO data set (P-value = 0.011, see Table S1). On the other hand, ProQ3 performs significantly better than all other non-ProQ methods on both data sets with P-values less than 0.05. The only exception is that the difference between ProQ3 and Dope is not significant on CAMEO per target correlations (P-value = 0.153).

#### Model selection

**Figure 6:**
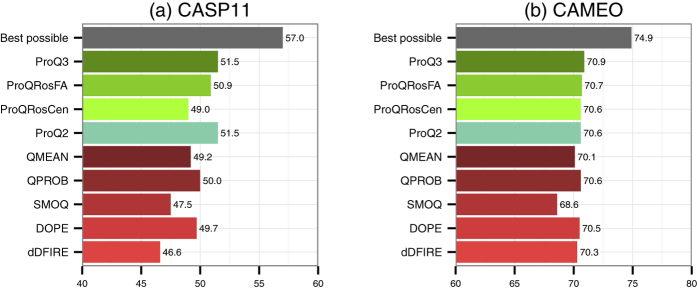
Average first ranked GDT_TS score for each method in the CASP11 and CAMEO data sets. Average is calculated over all targets in a data set.

An important task of MQA methods is to find the best protein model among several possible ones. We evaluated the performance of MQA methods in this aspect by calculating the average of first ranked GDT_TS scores for each method (see Fig. 6).

Interestingly, the original version of ProQ2 performs as well as ProQ3 in model selection. On CASP11 data set, they both have the average of first ranked GDT_TS score of 51.5 and outperform all other methods. Also here the differences are small in the CAMEO set due to the small variation in quality between the models.

We analysed the reasons of potential sub-optimal performance of ProQ3 in model selection and found that ProQ3 selects Robetta or other Rosetta-derived models more frequently than ProQ2, i.e. ProQ3 tends to over-estimate the quality of Rosetta models.

#### Using ProQ3 to re-rank models in structure prediction

In the CASP experiment, structure prediction groups can submit up to five models for each target and rank them from best to worst. The structure prediction groups are then evaluated by the sum or average of their first-ranked model scores. In CASP11^10^ concluded that some of the structure prediction groups could benefit from using ProQ2 in ranking their models. Similarly to their analysis, we evaluated how the average GDT_TS of the first ranked models would have changed for all structure prediction groups if they had been using ProQ3 and how this would have affected the group ranking (see Table S5).

We found that even the best structure prediction methods, except QUARK, would have benefited from using ProQ3. If Zhang-Server had been using ProQ3 to rank its models, it would have been ranked in first place (see Table 1). Moreover, BAKER_ROSETTASERVER would have jumped from the fifth to the second place.

#### Free modelling and template-based targets

The performance of MQA methods often differs depending on whether the data set consists of free modelling or template-based targets. Therefore, we decided to divide CASP11 targets into free modelling and template-based and evaluate all MQA methods on these data sets (see Tables S2 and S3). We have used the official CASP11 domain classification^28^. Targets with all domains classified as free modelling domains were classified as free modelling targets, while targets only template-based domains were considered template-based and all other targets were excluded from the evaluation.

On free modelling targets, ProQ3 outperforms all methods in global whole data set correlations, local whole data set correlations and local per model correlations (see Table S2), while ProQ2, QPROB and DOPE perform slightly better than ProQ3 in per target correlations and/or model selection. However, the number of targets in free modelling data set is rather small (15) and the mean model quality is very poor (S-score = 0.123), making it difficult to draw any firm conclusions. On template-based targets ProQ3 outperforms all other methods in all evaluation measures (Table S3).

## Conclusion

Here, we presented three novel model quality predictors: ProQRosFA, ProQRosCen and ProQ3. We show that these predictors by far outperform the original energy functions in Rosetta. The improved performance is mainly due to two factors: training SVM on individual energy terms and using different window sizes for averaging input features. After applying both of these strategies, the local (residue-level) correlation increase from 0.33/0.22 to 0.61/0.56 for ProQRosFA/ProQRosCen respectively.

We also include profile-based features: the agreement between predicted and observed RSA and SS values, as well as the conservation calculated from the profile directly. In the CASP11 data set, these three features alone reach a local correlation of 0.65 similar to the performance of the local predictors. By including these features into ProQRosFA/ProQRosCen predictors the correlation increase to 0.72/0.71 respectively. This correlation is on the same level as the original ProQ2 (0.72). Combining all three predictors into ProQ3 increases the local correlation to 0.74. In an independent set obtained from CAMEO, the correlation increases from 0.56 for ProQ2 to 0.62 for ProQ3 indicating the value of ProQ3.

In model quality assessment, the correlations between the predicted and target values can be calculated in several different ways: local vs. global, whole data set vs. per target vs. per model and model selection. All of these measures take into account different aspects of MQA performance and they are all relevant. We show that ProQ3 significantly outperforms ProQ2 in all of these different measures. ProQ2 has remained a superior single-model QA method since its introduction in 2012 even though several new single-model predictors were introduced later^10^,^21^,^22^. The improvement obtained by ProQ3 overall is small but significant. We also show that several different type of inputs provide similar performance and that the combination of them only provides a marginaly improvement. This might indicate that a radically different approach is needed to significantly enhance the performance of single model quality estimators.

ProQ3, ProQRosFA and ProQRosCen are all available as a webserver and as stand-alone programs (http://proq3.bioinfo.se/).

## Methods

### Training and test data sets

The original ProQ2 was trained on the CASP7 data set with 10 models per target selected at random. We noticed that the performance slightly increases when ProQ2 is retrained on the CASP9 data set with 30 models per target selected randomly. Therefore, we used the latter as the training data set for ProQRosFA, ProQRosCen and ProQ3.

Two data sets were used for testing: CASP11 and CAMEO. Only server models were used in the CASP11 data set. All CAMEO models from a time period of one year were used (2014–06–06–2015–05–30). Targets that were shorter than 50 residues were filtered out both from the CASP11 and CAMEO data sets. The CASP9 data set did not have such short targets.

Table 2 shows statistics of the data sets. We can see from the table that the CASP9 and CASP11 data sets have more models per target, but the CAMEO data set has more targets and the final number of models is in the same range in all data sets.

Mean model quality (S-score)) in the CASP9 and CASP11 data sets is similar (0.44 and 0.40), but in the CAMEO data set it is considerably higher (0.64). Mean standard deviation of model quality (calculated per target) in the CAMEO data set is much smaller (0.09).

### Target function

We used the same target function as in ProQ2, the S-score. The S-score is defined as:
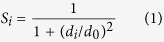
where *d*_*i*_ is the distance for residue *i* between the native structure and the model in the superposition that maximizes the sum of *S*_*i*_ and *d*_*0*_ is a distance threshold. The distance threshold was set to 3 Å, as in the original version of ProQ2.

### Side chain re-sampling and energy minimisation

Protein models can be generated by different methods that employ different modelling strategies resulting in similar models but vastly different Rosetta energy terms. For instance, some of models in our data sets had very large repulsive energy terms (fa_rep) because of steric clashes. To account for model generation differences, the side-chains of all models were rebuilt using the backbone-dependent rotamer library in Rosetta. This was followed by a short backbone restrained energy minimisation protocol (-ddg:min_cst) using the Rosetta energy function. This ensured that the Rosetta energy terms are minimized. ProQ3 performance is slightly better when side-chain repacking step is included (Table S4). Other MQA methods also use a similar side-chain repacking protocol to improve the performance^29^.

### ProQ3 run time

We evaluated ProQ3 run time on the CASP11 data set (Table 3). We ran ProQ3 in two modes: with and without side-chain repacking. As we discussed in the previous section, the side-chain repacking is necessary to avoid high repulsive energy terms because of steric clashes. However, the repacking step can be skipped if one is confident that the models have good quality side-chains without steric clashes.

Before running ProQ3, one has to run some external methods to generate profile-based features (solvent accessibility predictions, secondary structure predictions, residue conservation). The time to run these scripts depends mostly on the time it takes to run Psi-blast for the target sequence. Fortunately, Psi-blast has to be run only once per target sequence, because all models from one target shares the same sequence information.

ProQ3 was run using 1 CPU core, while Psi-blast was run using 8 CPU cores. ProQ3 does not support parallelisation, but running different models on different CPU cores is efficient.

### Implementation

We used the *per_residue_energies* binary in Rosetta (2014 week 5 release) to get per residue energies for local full-atom and centroid energy terms. *talaris2013.wts* weight file was used for local full-atom scoring function. For local centroid scoring function we defined a custom weight file that included *vdw*, *cenpack*, *pair*, *rama*, *env*, *cbeta* energy terms with all weights equal to one.

For global centroid scoring function, Rosetta *score* binary was used. A custom weight file included *rg*, *hs_pair*, *ss_pair*, *sheet*, *rsigma* and *co* energy terms with all weights equal to one.

SVM predictor works best when the input features are either scaled between −1 and 1 or between 0 and 1^30^. This is usually achieved by linear scaling of the input features. However, in order to avoid outliers we decided to use a sigmoidal function (1/(1 + *e*^*x*^)) to scale all of the terms between 0 and 1.

After the sigmoidal transformation, all of the local full-atom and centroid energy terms were averaged using window sizes of 5, 11 and 21 residues. Additionally, the local (single-residue) and the entire-model (averaged over the whole protein) energy terms were added to the training.

Global centroid energy terms are defined for the whole protein, so they cannot be averaged using different window sizes. On the other hand, they depend on the protein size, so they need to be normalised. *rg* term depends on the protein size L by a factor of *L*^0.4^ 31 by which it was normalised. After performing a linear regression on the logarithmic scale we found that *co* depends on the protein size by *L*^0.72^ and the other terms by L, so they were normalised accordingly.

### Profile-based features

The profile-based features, RSA, SS and Cons were implemented the same way as in ProQ2. Sequence profiles were derived using three iterations of PSI-BLAST v.2.2.26^32^ against Uniref90 (downloaded 2015–10–02)^33^ with an E-value inclusion threshold of 10^−3^. Secondary structure of the protein was calculated using STRIDE^34^ and predicted from the sequence profile using PSIPRED^35^. The agreement between the prediction and the actual secondary structure in the model was calculated over the window of 21 residues and over the entire model. Also, the probability of having a particular secondary structure type in every single position was calculated. Relative surface area accessibility was calculated by NACCESS^36^ and predicted from the sequence profile by ACCpro^37^. The RSA agreement was also calculated over the window of 21 residues and over the entire model. The actual secondary structure and relative surface area was not added to ProQRosFA and ProQRosCen predictors, only the agreement scores. For residue conservation “information per position” scores were extracted from PSI-BLAST matrix. The conservation for the central residue and two neighbouring residues was included into the SVM training.

### SVM training

A linear SVM model was trained using *SVM*^*light*^ package V6.02^38^. All parameters were kept at their default values.

### Running other methods

We ran QMEAN, Qprob, SMOQ, DOPE, dDFIRE with default parameters. The global score for DOPE method was derived in the same way as for ProQ methods—by summing up the local scores and dividing by the length of the target protein. The global scores for QMEAN and SMOQ were taken from the output as they are provided. Finally, Qprob and dDFIRE only provide the global scores, so they were only evaluated in the global evaluation category.

### Correlation calculation

We used Spearman rank correlation throughout in this paper.

### Other tools

R *zoo* package^39^ was used to average values over varying window sizes. The *needle* program from EMBOSS package^40^ was used to align model and target sequences.

## Additional Information

**How to cite this article**: Uziela, K. *et al*. ProQ3: Improved model quality assessments using Rosetta energy terms. *Sci. Rep*. **6**, 33509; doi: 10.1038/srep33509 (2016).

## Supplementary Material

Supplementary Information

## Figures and Tables

**Table 1:** Average GDT_TS1 for each method before and after re-ranking for top 5 prediction groups.

	Original GDT_TS1	ProQ3 GDT_TS1	ProQ2 GDT_TS1	Optimal GDT_TS1
QUARK	51.0	50.7	50.8	53.2
Zhang-Server	50.7	51.5	50.7	53.4
nns	49.7	49.7	49.8	51.7
myprotein-me	49.4	50.0	49.6	52.6
BAKER-ROSETTASERVER	49.2	50.8	50.7	53.2

**Table 2:** Training and test data sets.

	CASP9	CASP9 random subset	CASP11	CAMEO
Number of targets	117	117	83	676
Total number of models	33,440	3,505	15,334	20,206
Total number of residues	6,757,370	712,751	3,665,828	5,027,933
Average number of models per target	286	30	185	30
Average number of residues in a model	202	203	239	249
Mean model quality (S-score)	0.44	0.44	0.40	0.64
Mean per target standard deviation of model quality (S-score)	0.14	0.14	0.12	0.09

**Table 3:** ProQ3 run time on the CASP11 data set. The number of targets and models are the same as in Table 2.

	Total time	Time per target	Time per model
ProQ3-no-repack	3d 1 h 45 s	52 m 47 s	17 s
ProQ3-repack	15d 15 h 53 m 39 s	4 h 31 m 44 s	1 m 28 s
ProQ3-psiblast	15 h 21 m 29 s	11 m 6 s	—
