# Supplementary material for: ProQ3: Improved model quality assessments using Rosetta energy terms
Source: Sci Rep. 2016 Oct 4;6:33509. doi: 10.1038/srep33509 (PMC5048106; doi:10.1038/srep33509)
Supplement: Supplementary Information [file srep33509-s1.pdf]

Supporting material for: ProQ3: Improved model quality assessments using Rosetta energy terms

Karolis Uziela, Nanjiang Shu, Björn Wallner and Arne Elofsson

Table S1: **Wilcoxon test p-values for significance of differences in per target correlations between ProQ3 and other methods.** P-values lower than 0.05 are marked in bold.

	**ProQ2**	**ProQ-RosFA**	**ProQ-RosCen**	**QMEAN**	**QPROB**	**SMOQ**	**DOPE**	**DDFIRE**
**PROQ3, CASP11**	0.485	0.498	0.153	**3.91E-04**	**0.018**	**3.35E-09**	**8.25E-13**	**2.81E-09**
**PROQ3, CAMEO**	0.12	0.232	**0.011**	**9.13E-12**	**0.028**	**5.18E-46**	0.104	**0.019**

Table S2: **Method performance on CASP11 free modelling targets (15 targets).** Only targets that have all domains classified as free modeling domains according to the official CASP classification are considered.

	**Global whole data set correlation**	**Global per target correlation**	**Local whole data set correlation**	**Local per model correlation**	**Average first ranked GDT_TS**
**ProQ3**	0.520	0.492	0.334	0.285	21.9
**ProQRosFA**	0.475	0.471	0.305	0.272	21.9
**ProQRosCen**	0.470	0.482	0.279	0.206	20.5
**ProQ2**	0.507	0.506	0.298	0.244	21.9
**QMEAN**	0.446	0.474	0.231	0.206	21.8
**QPROB**	0.420	0.514	-	-	22.3
**SMOQ**	0.238	0.465	0.165	0.157	21.4
**DOPE**	0.224	0.371	0.037	0.082	22.5
**DDFIRE**	-0.366	0.383	-	-	17.7

Table S3: **Method performance on CASP11 template-based targets (52 targets).** Only targets that have all domains classified as template-based domains according to the official CASP classification are considered.

	**Global whole data set correlation**	**Global per target correlation**	**Local whole data set correlation**	**Local per model correlation**	**Average first ranked GDT_TS**
**ProQ3**	0.804	0.581	0.702	0.548	62.7
**ProQRosFA**	0.775	0.564	0.674	0.530	61.7
**ProQRosCen**	0.732	0.529	0.638	0.461	59.3
**ProQ2**	0.794	0.558	0.688	0.523	62.3
**QMEAN**	0.689	0.479	0.580	0.468	59.3
**QPROB**	0.717	0.483	-	-	60.1
**SMOQ**	0.501	0.329	0.394	0.295	57
**DOPE**	0.491	0.305	0.211	0.189	59.5
**DDFIRE**	0.306	0.369	-	-	57.2

Table S4: **ProQ3 performance with and without side-chain repacking.**

	**Global whole data set correlation**	**Global per target correlation**	**Local whole data set correlation**	**Local per model correlation**	**Average first ranked GDT_TS**
**ProQ3-no-repack, CASP11**	0.849	0.467	0.730	0.471	51.2
**ProQ3-repack, CASP11**	0.865	0.548	0.739	0.476	51.5
**ProQ3-no-repack, CAMEO**	0.725	0.322	0.600	0.503	69.6
**ProQ3-repack, CAMEO**	0.737	0.529	0.615	0.511	70.9

Table S5: **Average GDT_TS1 for each method before and after re-ranking for all prediction groups.** Targets column denotes on how many targets each group was evaluated. Groups that submitted only 1 model per target were excluded.

	Original GDT_TS1	ProQ3 GDT_TS1	ProQ2 GDT_TS1	Optimal GDT_TS1	Targets
**QUARK**	51.0	50.7	50.8	53.2	83
**Zhang-Server**	50.7	51.5	50.7	53.4	83
**nns**	49.7	49.7	49.8	51.7	83
**myprotein-me**	49.4	50.0	49.6	52.6	83
**BAKER-ROSETTASERVER**	49.2	50.8	50.7	53.2	83
**MULTICOM-CONSTRUCT**	48.9	48.4	48.5	50.5	83
**MULTICOM-CLUSTER**	48.8	49.3	49.2	50.2	83
**MULTICOM-NOVEL**	47.9	48.2	48.1	50.7	83
**RaptorX**	47.5	47.8	47.7	48.5	82
**HHPredA**	47.2	47.2	47.2	47.2	83
**HHPredX**	47.1	47.1	47.1	47.1	83
**FALCON_MANUAL_X**	47.0	47.2	47.3	48.1	83
**TASSER-VMT**	47.0	47.6	47.4	49.7	83
**FALCON_MANUAL**	47.0	47.0	47.2	48.2	83
**FALCON_TOPO**	46.9	47.0	46.9	48.0	83
**FALCON_EnvFold**	46.9	47.3	47.3	48.4	83
**Pcons-net**	46.3	46.8	46.2	48.4	83
**FFAS-3D**	46.3	46.8	46.7	47.7	83
**MULTICOM-REFINE**	46.2	46.0	46.1	47.5	83
**raghavagps-tsppred**	45.5	44.6	45.2	46.0	83
**PhyreX**	45.4	45.8	45.8	46.6	83
**BioSerf**	45.4	45.3	45.1	48.0	80
**Seok-server**	44.7	44.7	44.7	44.9	83
**chuo-fams-server**	43.5	43.7	44.0	45.3	83
**FLOUDAS_SERVER**	42.8	42.7	42.7	44.4	81
**FFAS03**	42.6	43.0	43.0	43.9	83
**eThread**	42.5	43.4	42.9	46.0	83
**slbio**	42.3	43.0	42.8	45.1	82
**Distill**	42.2	42.4	42.4	44.5	81
**3D-Jigsaw-V5_1**	42.1	42.2	42.2	43.9	82
**STRINGS**	42.0	43.6	42.8	46.3	81
**ZHOU-SPARKS-X**	41.7	42.2	42.3	44.9	83
**Atome2_CBS**	41.7	43.6	43.7	46.3	82
**RBO_Aleph**	41.4	42.0	41.9	44.3	81
**MUFOLD-Server**	41.2	41.2	41.2	42.5	83
**Alpha-Gelly-Server**	40.7	44.1	44.0	45.6	83
**BhageerathH**	38.7	40.7	40.6	41.7	83
**FUSION**	34.1	35.0	34.7	37.3	83
**BioShell-server**	32.9	34.3	34.6	36.9	81
**SAM-T08-server**	29.0	28.6	28.7	45.7	54
**PSF**	20.3	20.3	20.3	33.1	51
**MATRIX**	18.7	18.7	18.5	30.7	52
**IntFOLD3**	15.3	15.3	15.3	35.4	36
**RaptorX-FM**	8.0	8.3	8.4	23.2	32
